# Incidence, Predictors, and Outcomes Associated with Recurrent Neonatal Acute Kidney Injury in Preterm Infants: A Secondary Analysis of the Preterm Erythropoietin Neuroprotection Trial (PENUT)

**DOI:** 10.21203/rs.3.rs-10386644/v1

**Published:** 2026-08-03

**Authors:** Katherine Vincent, Austin D Rutledge, Tara Beck, Matthew C Gillen, Russell Griffin, Aadil Kakajiwala, Semsa Gogcu, Thomas Forbes, Michelle Starr, Judit Kiss, Shina Menon, David T Selewski, Cara L Slagle, Heidi J Steflik

**Affiliations:** Medical University of South Carolina; Medical University of South Carolina; University of Pittsburgh Medical Center Health System: University of Pittsburgh Medical Center; Emory University School of Medicine; University of Alabama at Birmingham; Children's National Medical Center: Children's National Hospital; Brown University; Royal Children's Hospital Research Institute: Murdoch Childrens Research Institute; Riley Hospital for Children: Riley Hospital for Children at Indiana University Health; University of Szeged: Szegedi Tudomanyegyetem; Stanford University School of Medicine; Medical University of South Carolina; Riley Hospital for Children: Riley Hospital for Children at Indiana University Health; Medical University of South Carolina

**Keywords:** Recurrent acute kidney injury, Neonatal acute kidney injury, Extremely low gestational age newborns, KDIGO, Prematurity, Neonatal outcomes

## Abstract

**Background:**

Recurrent neonatal acute kidney injury (rAKI) may increase morbidity and mortality but remains understudied. We aimed to determine the incidence, risk factors, and clinical outcomes associated with rAKI in extremely low gestational age newborns (ELGANs), hypothesizing that rAKI would be associated with worse outcomes than no AKI or single AKI (sAKI).

**Methods:**

We performed a secondary analysis (ALMOND study) of the multicenter Preterm Erythropoietin Neuroprotection (PENUT) Trial conducted from 2013–2016. AKI was diagnosed retrospectively using modified neonatal Kidney Disease: Improving Global Outcomes serum creatinine criteria. Outcomes included length of stay (LOS), bronchopulmonary dysplasia (BPD), mortality, and chronic kidney disease (CKD) and hypertension (HTN) at 2 years. Comparisons among no AKI, sAKI, and rAKI groups used multivariable generalized estimating equation regression and Cox proportional hazards models.

**Results:**

Among 923 ELGANs, 211 (23%) developed rAKI. Risk factors included younger gestational age, lower birthweight, small for gestational age, lower Apgar scores, patent ductus arteriosus, severe sepsis, nephrotoxic medication exposure, hypotension, BPD, and necrotizing enterocolitis (all *p* < 0.05).

## INTRODUCTION

Neonatal acute kidney injury (AKI) is associated with adverse hospital outcomes including increased mortality, length of stay (LOS), and duration of mechanical ventilation. [1-10] A new meta-analysis demonstrated that AKI in preterm infants is common with up to 18% prevalence in neonatal intensive care units (NICU).[11] Additionally, survivors of neonatal AKI are also at increased risk of developing chronic kidney disease (CKD) and hypertension (HTN). [12-15] Substantial efforts to better understand unique aspects of neonatal AKI in infants are underway due to its high prevalence, and short- and long-term consequences.

Infants, especially those born preterm, admitted to the neonatal intensive care unit (NICU) are subject to prolonged hospitalizations and are at risk for recurrent episodes of AKI (rAKI). Compared to a single episode of AKI (sAKI), rAKI is associated with increased morbidity and mortality. [16-18] In particular, rAKI has been shown to be associated with increased length of mechanical ventilation and LOS when compared to sAKI.[18] This suggests that rAKI may be a distinct clinical indicator of kidney health in infants. Available literature suggests that younger gestational age (GA) and lower birthweight (BW) are risk factors for rAKI. Extremely low gestational age newborns (ELGANs), born < 28 weeks of gestation are especially vulnerable to rAKI due to prolonged NICU stays, frequent comorbidities, and recurrent nephrotoxin exposure. Reported rAKI incidence in ELGANs ranges from 17% to 30% [16-18]. Despite this high incidence, multicenter data examining associations between rAKI and adverse outcomes in ELGANs remain limited.

To address these knowledge gaps, we performed a secondary analysis of the Preterm Erythropoietin Neuroprotection Trial (PENUT; ClinicalTrials.gov number NCT01378273). PENUT was a multi-center, randomized, placebo-controlled study of erythropoietin treatment of preterm infants 24 – 0/7 to 27 – 6/7 weeks gestation to decrease the combined outcome of death or neurodevelopmental impairment. We used kidney specific data from PENUT’s ancillary study, the Recombinant Erythropoietin for Protection of Infant Renal Disease (REPaIReD), which included long-term kidney outcomes on 85% of the PENUT subjects at 22 to 26 months corrected age.[19, 20] This database captured detailed kidney function data of ELGANs during NICU hospitalization and outpatient follow-up. We aimed to 1) describe the incidence of rAKI in ELGAN’s, 2) understand risk factors for rAKI, and 3) determine associations of rAKI with clinical short- and long-term outcomes. We hypothesized that ELGANs with rAKI would experience worsened short- and long-term outcomes compared to ELGANs with sAKI or no AKI episodes.

## METHODS

### Study Population

**Figure 1:**
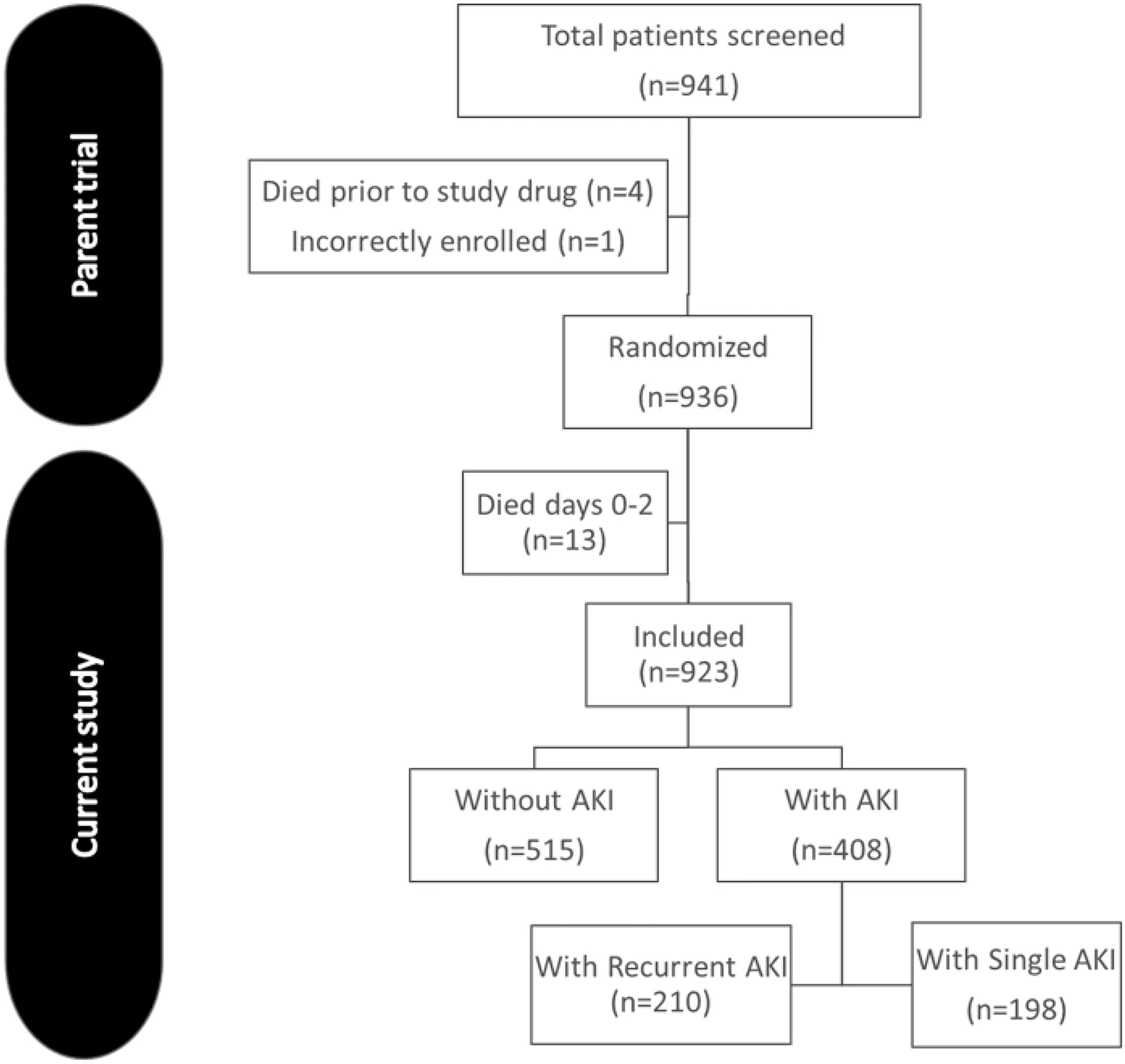
Study Enrollment Diagram Current study is secondary analysis of the Preterm Erythropoietin Neuroprotection Trial (PENUT)[19]. Abbreviations: AKI=acute kidney injury.

The PENUT trial (ClinicalTrials.org NCT01378273) included 941 infants born at 24 0/7 to 27 6/7 weeks’ gestation randomized to receive placebo or erythropoietin within 24 hours of birth.[19] Patients were excluded from the study if they had known life-threatening anomalies, chromosomal anomalies, disseminated intravascular coagulopathy, twin-to-twin transfusion, a hematocrit level above 65%, hydrops fetalis, or known congenital infection (Fig. 1).

All participating sites obtained approval from their respective IRBs or human research ethics committees before initiating the research. Written informed consent was secured from each infant’s parent or legal guardian. This current study is a *post hoc* analysis performed in September 2024 and was conducted and reported in accordance with the Strengthening the Reporting of Observational Studies in Epidemiology (STROBE) guidelines.[21] In addition to the exclusions of the PENUT study, we also excluded patients with less than four serum creatinine (SCr) measurements.

### Data Evaluation

A detailed description of the data collection methods for PENUT are previously published. [22] For this study, we evaluated baseline characteristics including GA, BW, Apgar scores, maternal characteristics, race, and ethnicity. Potential risk factors for rAKI were defined *a priori* and included patent ductus arteriosus (PDA), sepsis, nephrotoxic medication exposure, small for gestational age (SGA) status, vasopressor support, necrotizing enterocolitis (NEC), and severe intraventricular hemorrhage. All SCr values throughout NICU hospitalization were evaluated. Both short- and long-term outcomes including mortality, LOS, incidence of broncho-pulmonary dysplasia (BPD), CKD, and HTN were obtained.

### Definition of AKI and Recurrent AKI

The neonatal, modified Kidney Diseases Improving Global Outcomes (KDIGO) SCr criteria was used to identify and stage all AKI episodes.[23] Consistent with previous studies using REPaIReD data, AKI was defined using the following criteria: (1) baseline SCr was established as the lowest previously recorded value, excluding SCr levels obtained on the day of birth and on first postnatal day; (2) a SCr level of at least 0.5 mg/dL was required to meet diagnostic criteria; (3) evaluation began on postnatal day 3 to allow for the transient elevation in SCr during the first 48–72 hours of life attributable to maternal SCr levels;[23] and (4) at least four SCr levels per infant were required to be included in the study.

Recurrent AKI episodes were diagnosed only after SCr returned to the lowest previous baseline value (e.g., the SCr value utilized to diagnose the prior AKI episode), and the patient met AKI criteria again. Recurrent AKI staging was based on the highest stage of any recurrent episode utilizing KDIGO AKI staging criteria.

### Outcomes

The primary outcome was in-hospital mortality. Secondary outcomes included short- and long-term outcomes. Short-term outcomes included LOS, defined as duration of hospitalization (days), and BPD (determined in PENUT study using the Neonatal Research Network definitions as neonates receiving respiratory support at 28 days of age) [24]. Long-term outcomes included CKD and HTN assessed at two years of age. CKD was defined as an estimated glomerular filtration rate (eGFR) < 90 ml/min/1.73 m^2^, defined by the combined SCr and cystatin C-based CKiD (Chronic Kidney Disease in children) equation. HTN was defined as systolic blood pressure values exceeding the 90th percentile for age-related and sex-related normative values[25]. Comparisons were made between those with no AKI, sAKI and rAKI.

### Statistical Analysis

Continuous data are presented as median [interquartile range, IQR] or mean ± standard deviation depending on their distribution. Categorical data are presented as frequencies (percentages). Demographic data, baseline characteristics, and potential risk factors were compared among those with no AKI, sAKI, and rAKI using Chi-square, Fisher’s exact, or analysis of variance (ANOVA) tests as appropriate. AKI episode severity was compared between those with sAKI and rAKI using Chi-square.

Cox proportional hazards regression models were used to estimate hazard ratios (HRs) and associated 95% confidence intervals (CIs) for the time-varying effect of AKI status on mortality, LOS, and incidence of BPD; models were stratified by study site to account for differing baseline hazards of the outcome at each site. The use of a time-varying exposure allowed subjects to transition between AKI categories, from no AKI to sAKI and from sAKI to rAKI status, as indicated. Final adjusted models were selected by utilizing a best-subset process based on the Akaike Information Criterion score[26, 27]. A Cox regression model assuming equal time at risk was used to estimate risk ratios (RRs) and associated 95% CIs for the associations between no AKI, sAKI, and rAKI and BPD, CKD, and HTN. As with the short-term outcome regression analysis, final adjusted models were selected by utilizing a best-subset process based on the Akaike Information Criterion score. A p-value of < 0.05 was determined a priori to be statistically significant for all analyses. All statistical analyses were performed using SAS (version 9.4; SAS Institute, Inc. Cary, NC).

## RESULTS

### Study Population

After screening the 941 infants who met inclusion criteria for PENUT, 936 infants were randomized and 923 infants ultimately included in this analysis (Figure 1). The mean BW was 801±188 grams (Table 1).

### Recurrent Acute Kidney Injury

**Figure 2:**
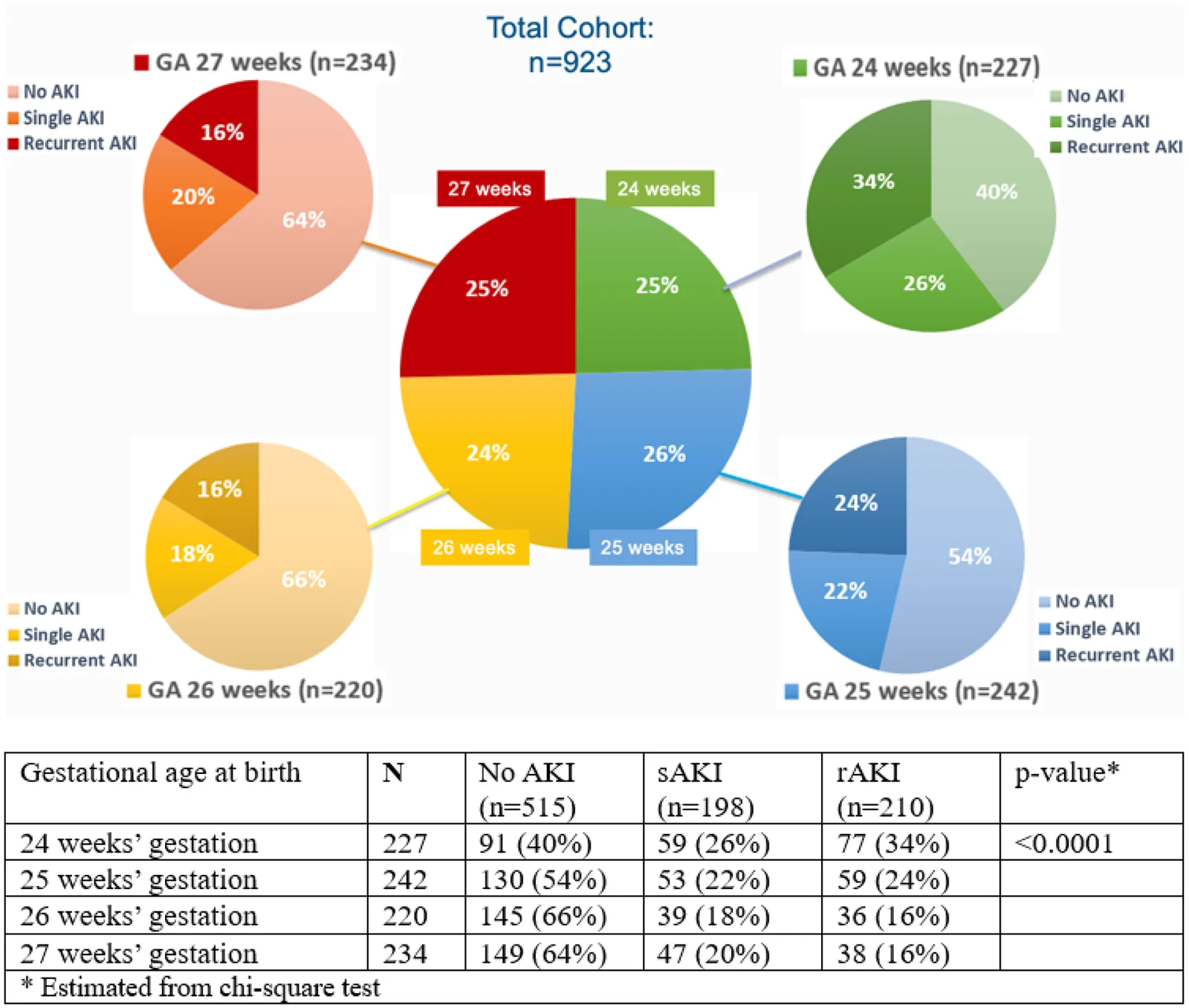
Acute Kidney Injury Stratified by Gestational Age

There was a total of 15,133 SCr measurements with a median of 31 (IQR 21-45) SCr measurements per patient for analysis. AKI occurred in 44% (N=408) of the cohort and rAKI occurred in 51% (N=210) of those with AKI (23% of the overall cohort). The rates of AKI differed by GA with infants born at 24 weeks’ gestation experiencing the highest rates of rAKI (34%; Figure 2). Infants who experienced rAKI had significantly lower BW and were more frequently SGA compared to infants with sAKI or no AKI (Table 1). Additionally, those with rAKI experienced significantly lower 1- and 5-minute Apgar scores compared to infants with sAKI or no AKI (Table 1).

Infants with rAKI experienced more severe AKI than infants with sAKI: 38% of infants with rAKI experienced stage 2 AKI compared to 24% of sAKI infants. Twenty-five percent of infants with rAKI experienced stage 3 AKI compared to 9% of infants with sAKI (Table 2a). In those who developed rAKI, 38% of rAKI episodes were stage 1 AKI and 62% of rAKI episodes were stage 2 or 3. The second episode of AKI was diagnosed at median 42 (IQR 25,74) days of life and 27 days after the initial AKI episode (Table 2b).

### Risk Factors Associated with Recurrent Acute Kidney Injury

Known neonatal AKI risk factors were more prevalent in infants with rAKI compared to those with sAKI or no AKI, including PDA, sepsis, exposure to vancomycin, hypotension, and NEC (Table 1).

### Recurrent Acute Kidney Injury and Short-Term Outcomes: Associations with In-Hospital Mortality, Length of Stay, and Bronchopulmonary Dysplasia

**Figure 3:**
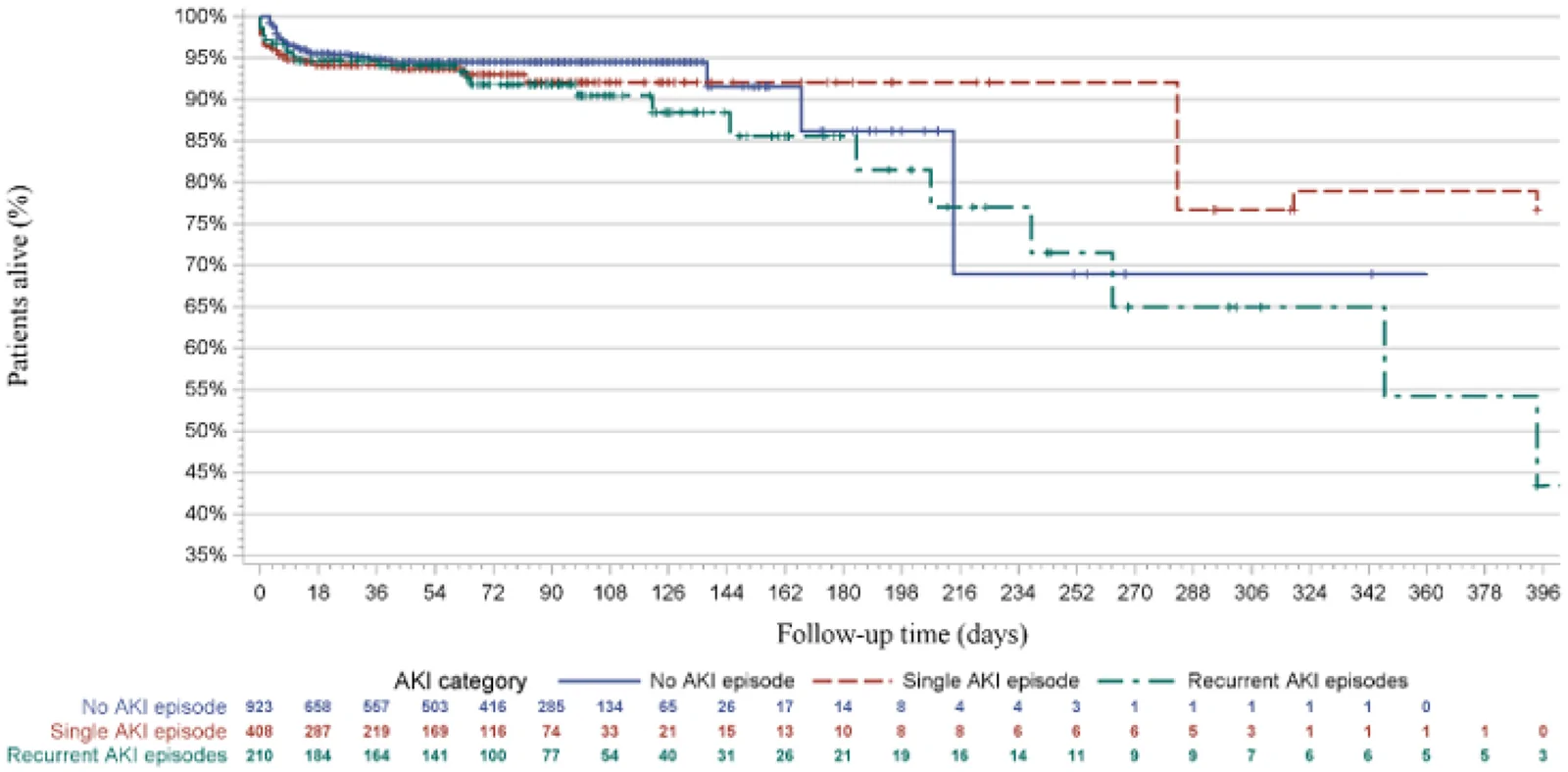
Kaplan-Meier Curve[AR1] : Mortality Over Time

After adjusting for multiple potential confounders, we found a significant increased hazard of mortality among those with rAKI (aHR 2.79, 95% CI, 1.05-7.46, p=0.04) when referencing no AKI. There was no difference in mortality when comparing rAKI to sAKI (Table 3). However, a Kaplan Meier curve demonstrates overall, as time progressed, fewer patients with rAKI survived (Figure 3).

Mean LOS for the entire cohort was 94.6±58.3 days. Infants with rAKI had longer median LOS (108 [IQR 85-139] days) than those with sAKI (93 [IQR 71-114] days) or no AKI (88 [IQR 70-105] days) p<0.01. After adjusting for multiple potential confounders, infants with rAKI experienced longer LOS evidenced by a lower hazard of discharge when compared to infants with no AKI (aHR 0.58, 95% CI 0.45-0.74, p<0.01) and infants with sAKI (aHR 0.71, 95% CI 0.56-0.89, p<0.01) (Table 3).

BPD was common in this cohort of ELGANs. In total, 341 infants (37%) developed BPD, and the rate of BPD was highest in those with rAKI (108, 51%). However, in multivariable analysis, we did not detect an association with rAKI and BPD (Table 3).

### Recurrent Acute Kidney Injury and Long-Term Outcomes: Associations with Chronic Kidney Disease and Hypertension

Approximately half of the enrolled infants had data available until two years of age to evaluate long-term outcomes including CKD (n=336 evaluated, 36%) and HTN (n=492 evaluated, 53%). CKD was diagnosed in 54 (16%) children, and HTN was present in 290 (59%). We did not detect any association between rAKI and development of CKD or HTN (Table 4).

## DISCUSSION

In this large cohort of ELGANs, rAKI occurred in 23% of the overall cohort and in 51% of those who developed any AKI, highlighting the increased prevalence of rAKI in premature infants. Infants with rAKI had more severe AKI episodes (stage 2 or 3) compared to infants with sAKI suggesting that recurrent episodes may cause incremental harm to the kidney. Infants with rAKI also had increased mortality compared to those with no AKI. However, when comparing rAKI to sAKI there was no difference in mortality which could reflect survival bias, as an infant must continue to survive in order to develop rAKI. Infants with rAKI also had longer adjusted LOS compared to those with no AKI or sAKI, which places these infants at increased risk for additional morbidities associated with hospitalization and creates a financial burden to the healthcare system[28].

Little is known about the incidence, risk factors or outcomes of rAKI in infants. Single center studies and a solitary multicenter study suggest neonatal rAKI is associated with increased LOS and mortality, but the impacts of rAKI vary with GA [16-18]. While our study contributes to this growing body of literature and demonstrates associations between rAKI and LOS in particular, causation cannot be inferred. ELGANs experience long hospital admissions and are often repeatedly exposed to AKI risk factors including sepsis, hypotension, nephrotoxic medications, fluid imbalance, and surgical interventions [29, 30], increasing their vulnerability to rAKI. However, it is also possible that the longer hospitalization allows for increased monitoring of SCr and identification of rAKI episodes. In our data set, we interestingly noted that among those with rAKI, the more severe stage 2 and 3 recurrent episodes of AKI occurred earlier in the hospital course than the stage 1 recurrent episodes of AKI; this suggest that perhaps severe AKI early on predisposes to recurrent, though milder, episodes of AKI in the future. Larger future studies are needed to more clearly understand this association.

This detailed, multicenter dataset allowed the study of rAKI in a large cohort of ELGANs and analyzed short and long-term outcomes of rAKI, up to two years of age. Our findings are also consistent with Rutledge et al.'s secondary analysis of the Assessment of Worldwide Acute Kidney Injury Epidemiology in Neonates (AWAKEN) study, an analysis assessing the impact rAKI has on infants of all gestational ages[17]. Rutledge found a similar incidence of rAKI (22% vs 23%) in infants with similar key risk factors of rAKI such as younger GA and lower BW [17]. Both studies found an association between rAKI and longer LOS. However, unlike Rutledge et al. who only found increased mortality in infants with rAKI that were greater than 36 weeks’ GA, we found that rAKI was independently associated with increased mortality in ELGANs, suggesting that this group may be especially vulnerable to the impact of recurrent injury despite the potential for survival bias [17]. Additionally, this sample size of ELGANS is much higher in our study compared with AWAKEN (n = 273 for GA < 29 weeks). The first study, to our knowledge, of rAKI in neonates was a retrospective evaluation by Adegboyega et al. in 205 ELGANs used a similar diagnostic scheme with KDIGO staging and reported lower rates of rAKI (24%) than this analysis, but similar significant associations between rAKI and mortality as well as extended LOS[16]. Interestingly, they found differences in mortality between those with rAKI compared to those with sAKI, while we only demonstrated differences between those with rAKI compared to those without AKI. Similarly, they also found associations between rAKI and increased LOS though their effect size what larger than ours (36 vs. 15 days). There are several explanations about the differences in findings, including potential differences in SCr monitoring, however these general findings reiterate the importance of recognizing and diagnosing rAKI.

Our findings did not demonstrate differences in long-term outcomes like BPD, CKD, and HTN between sAKI and rAKI groups at age two. It is possible that prematurity alone is a stronger underlying risk factor for CKD and therefore AKI may not add much additional risk. Previous literature suggests HTN may present even later in childhood beyond our 2 years[31, 32]. Therefore, it is possible that longer periods of follow-up are needed to capture associations with these more childhood onset chronic morbidities. Overall, our findings reinforce that rAKI is a clinically meaningful event in ELGANs and highlight the need for early identification and prevention strategies.

The strengths of this study include the use of a large, dataset from a multicenter, randomized, placebo-controlled study with detailed SCr and outcome data. To our knowledge, this the largest evaluation of rAKI in preterm infants to date. The inclusion and exclusion as well as the retrospective data collection of the PENUT study dataset introduces some limitations. PENUTs exclusion criteria may have potentially excluded patients that may have been affected by AKI or rAKI. While the data collection was retrospective data points used in this re-analysis were largely objective and all SCr measurements were manually reviewed and adjudicated to determine AKI and rAKI. Another limitation is that urine output was not assessed due to the difficulty in accurately measuring urine output in infants and thus some AKI episodes could have been missed. While the modified KDIGO criteria is the gold standard for diagnosing AKI, it can over or underestimate AKI episodes. Per the criteria, AKI is defined as 1.5 times baseline and therefore a small increase in SCr from 0.2 mg/dL to 0.3 mg/dL would define as stage 1 AKI, which may be overestimating AKI episodes in infants; however, we found stage 2 and stage 3 AKI to be more common in the rAKI cohort. The criteria also defines rAKI as a complete return of SCr to baseline before defining a new AKI episode, which may underestimate the incidence of rAKI in circumstances where an episode of rAKI occurred before full biochemical recovery.

## CONCLUSION

In this multicenter study of ELGANs, we show that rAKI in the NICU is common and is independently associated with increased mortality and longer LOS. Future studies into childhood are needed to determine if CKD and HTN are associated with rAKI. In summary, these results support the findings in previous studies and suggest rAKI is a clinically important occurrence and a distinct morbidity warranting special attention and further study.

## Supplementary Material

This is a list of supplementary files associated with this preprint. Click to download.

• STROBEchecklist.docx

## Figures and Tables

**Table 1.** Comparison of Neonatal, Maternal and Acute Kidney Injury (AKI) Factors Among Acute Kidney Injury Classifications

	Total Cohort (n=923)	No AKI (n=515)	Single AKI (n=198)	Recurrent AKI (n=210)	*p-*value*
**NEONATAL CHARACTERISTICS**					
Erythropoietin, yes	469 (50.8)	264 (51.1)	101 (60.8)	104 (49.3)	0.91
Mean Birth weight, grams	801±188	832±187	782±188	744±175	**<0.01**
Small size for gestational age	141 (15.2)	66 (12.8)	32 (16.1)	43 (20.6)	**0.03**
Mean Apgar 1-minute	3.9±2.3	4.1±2.3	3.8±2.3	3.6±2.2	**0.01**
Mean Apgar 5-minute	6.2±2.1	6.4±1.9	5.9±2.3	5.9±2.2	**<0.01**
Delivery room resuscitation					
Any	896 (97)	497 (96.7)	192 (97.0)	207 (98.6)	0.38
Intubation	748 (81)	399 (76.9)	166 (83.4)	183 (87.2)	**<0.01**
Surfactant	480 (52)	241 (46.8)	113 (57.1)	126 (60.0)	**<0.01**
Chest compressions	72 (7.8)	41 (7.9)	19 (9.5)	12 (5.7)	0.34
Resuscitation drugs	32 (3.5)	15 (2.9)	8 (4.0)	9 (4.3)	0.58
**MATERNAL CHARACTERISTICS**					
Multiple gestations	243 (26.3)	131 (25.4)	56 (28.3)	56 (26.7)	0.74
Diabetes	48 (5.2)	30 (5.8)	7 (3.5)	11 (5.2)	0.47
Hypertension	70 (7.6)	31 (6.0)	18 (9.0)	21 (10.0)	0.12
Pre-eclampsia	140 (15.2)	78 (15.1)	29 (14.6)	33 (15.7)	0.96
Mean Maternal age	28.9±6.2	29.3±6.0	28.8±6.4	28.0±6.5	**0.02**
Maternal Race					
Black	239 (25.9)	132 (25.6)	42 (21.2)	65 (31.0)	
White	604 (65.4)	341 (66.2)	139 (70.2)	124 (59.0)	0.17
Other/Unknown	80 (8.7)	42 (8.2)	17 (8.6)	21 (10.0)	
**RISK FACTORS FOR AKI**					
Patent ductus arteriosus	384 (41.6)	177 (34.4)	84 (42.6)	123 (58.6)	**<0.01**
Severe sepsis	78 (8.5)	26 (5.0)	24 (12.1)	28 (13.3)	**<0.01**
Nephrotoxic exposure					
NSAID	506 (54.8)	277 (53.8)	100 (50.5)	129 (61.4)	0.07
Gentamycin	882 (95.6)	488 (94.8)	191 (96.5)	203 (96.7)	0.41
Vancomycin	560 (60.7)	269 (52.2)	132 (66.7)	159 (75.7)	**<0.01**
Hypotension with pressor	376 (40.7)	163 (31.7)	91 (46.0)	122 (58.1)	**<0.01**
Necrotizing enterocolitis	99 (10.7)	29 (5.6)	30 (15.1)	40 (19.0)	**<0.01**
Severe IVH	122 (13.2)	61 (11.8)	28 (14.1)	33 (15.7)	0.34
**PROTECTIVE FOR AKI**					
Caffeine	914 (99)	511 (99.2)	195 (98.5)	208 (99.0)	0.67

Categorical variables presented as count (percentage). Continuous data presented as median ± standard deviation. NSAID, nonsteroidal anti-inflammatory drugs; IVH, intraventricular hemorrhage.

* Based on Pearson’s chi-square test or analysis of variance for categorical and continuous variables, respectively

**Table 2.** Incidence and Severity of Acute Kidney Injury (AKI) by KDIGO Stage

AKI Incidence and Severity	Single AKI (n=198)	Recurrent AKI (n=210)	*p-*value*
**KDIGO Stage**			
**Stage 1**	133 (67.2)	79 (37.6)	**<0.01**
**Stage 2**	47 (23.7)	79 (37.6)	
**Stage 3**	18 (9.1)	52 (24.8)	

Categorical variables presented as count (percentage).

* Estimated from chi-square test

KDIGO, Kidney Disease: Improving Global Outcomes[23].

**Table 2b.** Median and Interquartile Range of the Timing and Severity of Acute Kidney Injury (AKI), Overall and by AKI Stage

	Single AKI	Recurrent AKI	*p*-value*
**ALL AKIs**			
Initial AKI episodes, n	198	210	
Initial episode (DOL)	16 (9,32)	15 (7,27)	0.1304
Recurring episodes (DOL)	-	42 (25,74)	
			
**STAGE 1 AKIs**			
Initial AKI episodes, n	133	146	
Initial episode (DOL)	13 (7,28)	13 (6,29)	0.5480
Recurring episodes, n	-	79	
Recurring episodes (DOL)	-	51 (32,79)	
			
**STAGE 2/3 AKIs**			
Initial AKI episodes, n	65	64	
Initial episode (DOL)	19 (13,36)	18 (9,26)	0.0760
Recurring episodes, n	-	131	
Recurring episodes (DOL)	-	38 (21,65)	
			

* Estimated from a Wilcoxon rank sums test

AKI staged by KDIGO (Kidney Disease: Improving Global Outcomes) criteria[23].

**Table 3.** Crude and Adjusted Hazard Ratios (HRs) and Associated 95% Confidence Intervals (CIs) for the Association between Number of Acute Kidney Injury (AKI) Episodes and Mortality, Discharge from the Hospital (i.e., length of stay), and Bronchopulmonary Dysplasia (BPD)

AKI Episodes	N (%) or Median (IQR)	Crude HR* (95% CI)	*p-*value	Adjusted HR*†‡ (95% CI)	*p-*value
**In-Hospital MORTALITY**					
No AKI	47 /515 (9.1)	Referent		Referent	
Single AKI	27/198 (13.6)	2.59 (1.39-4.83)	**<0.01**	1.46 (0.73-2.92)	0.28
Recurrent AKI	26/210 (12.4)	4.32 (1.83-10.22)	**<0.01**	2.79 (1.05-7.46)	**0.04**
Recurrent vs. Single	-	1.67 (0.76-3.66)	0.2	1.91 (0.80-4.54)	0.14
**DISCHARGE (days)**					
No AKI	88 (70-105)	Referent		Referent	
Single AKI	93 (71-114)	0.64 (0.53-0.78)	**<0.01**	0.82 (0.67-1.00)	**0.05**
Recurrent AKI	108 (85-139)	0.40 (0.31-0.50)	**<0.01**	0.58 (0.45-0.74)	**<0.01**
Recurrent vs. Single	-	0.62 (0.49-0.78)	**<0.01**	0.71 (0.56-0.89)	**<0.01**
**BPD**					
No AKI	157/515 (30.5)	Referent		Referent	
Single AKI	76/198 (38.4)	1.26 (0.96-1.66)	0.1	1.03 (0.77-1.36)	0.86
Recurrent AKI	108/210 (51.4)	1.70 (1.33-2.17)	**<0.01**	1.24 (0.96-1.60)	0.11
Recurrent vs. Single	-	1.34 (1.00-1.79)	**0.05**	1.21 (0.90-1.63)	0.22

* Based on Cox proportional hazards model stratified by study site and including time-varying covariates for single and recurrent AKI

† Mortality model adjusted for maternal pre-eclampsia, surfactant and chest compressions used during resuscitation, NEC, PDA, severe sepsis, severe IVH, vasopressor exposure, vancomycin exposure, caffeine exposure, and 1-minute Apgar

‡ Discharge model adjusted for gestational age, maternal ethnicity, multiple gestation, gestational diabetes, small for gestational age, surfactant use during resuscitation, NEC, severe sepsis, NSAID exposure, BPD, vancomycin exposure, and 5-minute Apgar

¶ BPD model adjusted for gestational age, sex, gestational diabetes, intubation during resuscitation, use of surfactant during resuscitation, small for gestational age, PDA, severe sepsis, vancomycin exposure, and 1-minute Apgar

**Table 4.** Crude and Adjusted Hazard Ratios (HRs) and Associated 95% Confidence Intervals (CIs) for the Association between Number of Acute Kidney Injury (AKI) Episodes and Chronic Kidney Disease (CKD) and Hypertension at 2 years of age

AKI episodes	N (%)	Crude HR* (95% CI)	*p-*value	Adjusted HR*†‡ (95% CI)	*p-*value
**CKD**					
No AKI	26/182 (14.3)	Referent			
Single AKI	13/69 (18.8)	1.32 (0.68-2.57)	0.42	1.26 (0.64-2.48)	0.5
Recurrent AKI	15/85 (17.6)	1.22 (0.65-2.31)	0.38	1.06 (0.53-2.10)	0.88
Recurrent vs. Single	-	0.93 (0.44-1.95)	0.84	0.84 (0.39-1.82)	0.65
**HYPERTENSION**					
No AKI	163/276 (59.1)	Referent			
Single AKI	57/106 (53.8)	0.91 (0.67-1.23)	0.53	0.91 (0.67-1.24)	0.55
Recurrent AKI	70/110 (63.6)	1.08 (0.82-1.43)	0.59	1.12 (0.84-1.49)	0.44
Recurrent vs. Single	-	1.19 (0.84-1.69)	0.33	1.23 (0.86-1.74)	0.26

* Based on Cox proportional hazards model stratified by study site and including time-varying covariates for single and recurrent AKI

† CKD model adjusted for gestational age, pre-eclampsia, small for gestational age, chest compressions during resuscitation, severe sepsis, NSAID exposure, and maternal age

‡ Hypertension model adjusted for sex, ethnicity, PDA, and NSAID exposure
